# Combined ambient ionization mass spectrometric and chemometric approach for the differentiation of hemp and marijuana varieties of *Cannabis sativa*

**DOI:** 10.1186/s42238-023-00173-0

**Published:** 2023-02-18

**Authors:** Megan I. Chambers, Samira Beyramysoltan, Benedetta Garosi, Rabi A. Musah

**Affiliations:** Department of Chemistry, University at Albany, State University of New York (SUNY), 1400 Washington Avenue, Albany, NY 12222 USA

**Keywords:** *Cannabis sativa*, Ambient ionization mass spectrometry, Direct analysis in real time—high-resolution mass spectrometry, Multivariate data analysis, Random forest, Principal component analysis

## Abstract

**Background:**

Hemp and marijuana are the two major varieties of *Cannabis sativa*. While both contain Δ^9^-tetrahydrocannabinol (THC), the primary psychoactive component of *C. sativa*, they differ in the amount of THC that they contain. Presently, U.S. federal laws stipulate that *C. sativa* containing greater than 0.3% THC is classified as marijuana, while plant material that contains less than or equal to 0.3% THC is hemp. Current methods to determine THC content are chromatography-based, which requires extensive sample preparation to render the materials into extracts suitable for sample injection, for complete separation and differentiation of THC from all other analytes present. This can create problems for forensic laboratories due to the increased workload associated with the need to analyze and quantify THC in all *C. sativa* materials.

**Method:**

The work presented herein combines direct analysis in real time—high-resolution mass spectrometry (DART-HRMS) and advanced chemometrics to differentiate hemp and marijuana plant materials. Samples were obtained from several sources (e.g., commercial vendors, DEA-registered suppliers, and the recreational *Cannabis* market). DART-HRMS enabled the interrogation of plant materials with no sample pretreatment. Advanced multivariate data analysis approaches, including random forest and principal component analysis (PCA), were used to optimally differentiate these two varieties with a high level of accuracy.

**Results:**

When PCA was applied to the hemp and marijuana data, distinct clustering that enabled their differentiation was observed. Furthermore, within the marijuana class, subclusters between recreational and DEA-supplied marijuana samples were observed. A separate investigation using the silhouette width index to determine the optimal number of clusters for the marijuana and hemp data revealed this number to be two. Internal validation of the model using random forest demonstrated an accuracy of 98%, while external validation samples were classified with 100% accuracy.

**Discussion:**

The results show that the developed approach would significantly aid in the analysis and differentiation of *C. sativa* plant materials prior to launching painstaking confirmatory testing using chromatography. However, to maintain and/or enhance the accuracy of the prediction model and keep it from becoming outdated, it will be necessary to continue to expand it to include mass spectral data representative of emerging hemp and marijuana strains/cultivars.

**Supplementary Information:**

The online version contains supplementary material available at 10.1186/s42238-023-00173-0.

## Background

Among the greatest challenges to emerge for U.S. crime laboratories in recent years are those attributed to the increased legalization and decriminalization of marijuana at the state level, in addition to the permitted production of hemp. The 2019 National Institute of Justice (NIJ) “Report to Congress: Needs Assessment of Forensic Laboratories and Medical Examiner/Coroner Offices” identified this area as requiring focused attention towards improving criminal justice practices in the USA (NIJ 2019). The challenge that hemp and marijuana present is as follows: both are major varieties of the same species *Cannabis sativa*, often referred to as *Cannabis*. While they each contain Δ^9^-tetrahydrocannabinol (Δ^9^-THC), which is the primary psychoactive component of *C. sativa*, marijuana and hemp differ in the amount of this molecule that is present. In 2018, the U.S. federal guidelines stipulated that *C. sativa* which contains greater than 0.3% THC is a scheduled controlled substance (i.e., marijuana), while plant material that contains less than or equal to 0.3% is a legal agricultural commodity (i.e., hemp) (H.R.2 – 115th Congress 2017–2018). This definition has imposed severe challenges on crime labs. Among them is the dramatic increase in workload that results from the need to analyze and quantify the THC content of *all C. sativa* samples so that seized material can be appropriately designated. This is a time-consuming and resource-intensive enterprise that to greater and greater extents is consuming even larger crime lab resources. Furthermore, defining the error cutoff for the 0.3% designation presents a challenge for the analysis of samples whose THC level is at the threshold.

Traditionally, hemp and marijuana plant materials are differentiated by determining the THC content through chromatography-based approaches such as gas chromatography-flame ionization detection (GC-FID) and gas chromatography-mass spectrometry (GC–MS) (Pourseyed Lazarjani et al. 2020), in addition to high-performance liquid chromatography (HPLC) coupled to ultraviolet (UV) detection (UNODC 2009). However, to accurately determine the THC content with these approaches, THC must be separated from all other components in the material (i.e., cannabinoids, terpenes, etc.) prior to quantification. One way to achieve this is to extend run times to allow for baseline separation between cannabinoids and other analytes present. Another option is to introduce a chemical derivatization step into the sample preparation protocol (which can be time-consuming), to differentiate between cannabinoids and their corresponding cannabinoid acids (e.g., THC and THCA). Although many investigations have been successful at differentiating between hemp and marijuana varieties or strains (Wiebelhaus et al. 2016; Horne et al. 2020; Pacula et al. 2016; Fischedick et al. 2010), the methods are reliant upon chromatography and are therefore susceptible to the aforementioned delineated challenges that can arise using this technique (i.e., lengthy run times, column contamination, etc.). Research towards developing, optimizing, and validating methods suitable for field testing of *Cannabis* materials has also been investigated. Colorimetric tests represent a large percentage of these methods, which yield a presumptive result (by producing a color change) (Alonzo et al. 2018) when *Cannabis*-related substances are present, without the need for additional instrumentation (i.e., it is visible to the naked eye). Some examples include the 4-aminophenol test (Lewis et al. 2021; Acosta et al. 2022), Fast Blue BB test (Acosta et al. 2022; Acosta and Almirall 2021), and Duquenois-Levine test (Forrester 1997). Similar to chromatography-based methods, these tests all rely upon the detection of THC specifically, which can complicate analyses because both marijuana and hemp contain this compound. Thus, while the distinction between marijuana and hemp has been defined based on THC levels, this is accompanied by several analytical challenges (i.e., baseline separation of molecules by chromatography-based methods, lengthy sample preparation protocols, and presumptive tests that can yield false positives (Gabrielson et al. 2016), etc.).

An alternative less arbitrary approach is to base the distinction between them on the *genome-defined differences* in their metabolome signatures (i.e., small-molecule profiles). Studies utilizing the genetic profiles of *Cannabis*, such as genotyping-by-sequencing (GBS) and single-nucleotide polymorphisms (SNPs), have shown that, although they represent the same species, hemp and marijuana differ at the genome-wide level (Sawler et al. 2015; Roman et al. 2020; Schwabe et al. 2021). However, in addition to the fact that many crime laboratories are not positioned to integrate these types of analyses into current workflows, one of the bottlenecks to the routine use of the genome-defined small-molecule profiles for species attribution is the challenge of accessing this information quickly and reliably. One way to rapidly reveal this information, and subsequently distinguish between hemp and marijuana, is to combine an ambient ionization mass spectrometric technique (e.g., direct analysis in real-time—high-resolution mass spectrometry, or DART-HRMS) (Cody et al. 2005), with advanced statistical analysis. Ambient ionization methods (e.g., DART-HRMS, desorption electrospray ionization (DESI-MS)) have proven successful at screening for cannabinoids in *Cannabis* plant materials (Chambers and Musah 2022; Rodriguez-Cruz 2006; Chambers and Musah 2023) and *Cannabis*-derived products (e.g., edibles, personal-care products, vape products, concentrates) (Chambers and Musah 2022; Chambers and Musah. 2023). The unique capabilities of DART-HRMS are well-suited for the analysis of complex plant materials; the results are characterized by having high chemical information content, and little to no sample preparation prior to interrogating the materials is required. When applied to DART-HRMS-derived spectra, statistical data processing has enabled the successful differentiation of psychoactive plant species (Beyramysoltan et al. 2019) and their headspace chemical signatures (Appley et al. 2019). A modified version of DART-MS analysis introduced thermal desorption (TD) into the methodology (TD-DART-MS). One study utilized TD-DART-MS data to differentiate four hemp cultivars using PCA and partial least squares discriminant analysis (PLS-DA) (Dong et al. 2019). Another found that the application of statistical analysis to DART-MS data derived from methanolic extracts of hemp and marijuana samples revealed the potential for utilizing this method for optimally differentiating hemp and marijuana varieties (Pieslak 2021).

**Fig. 1:**
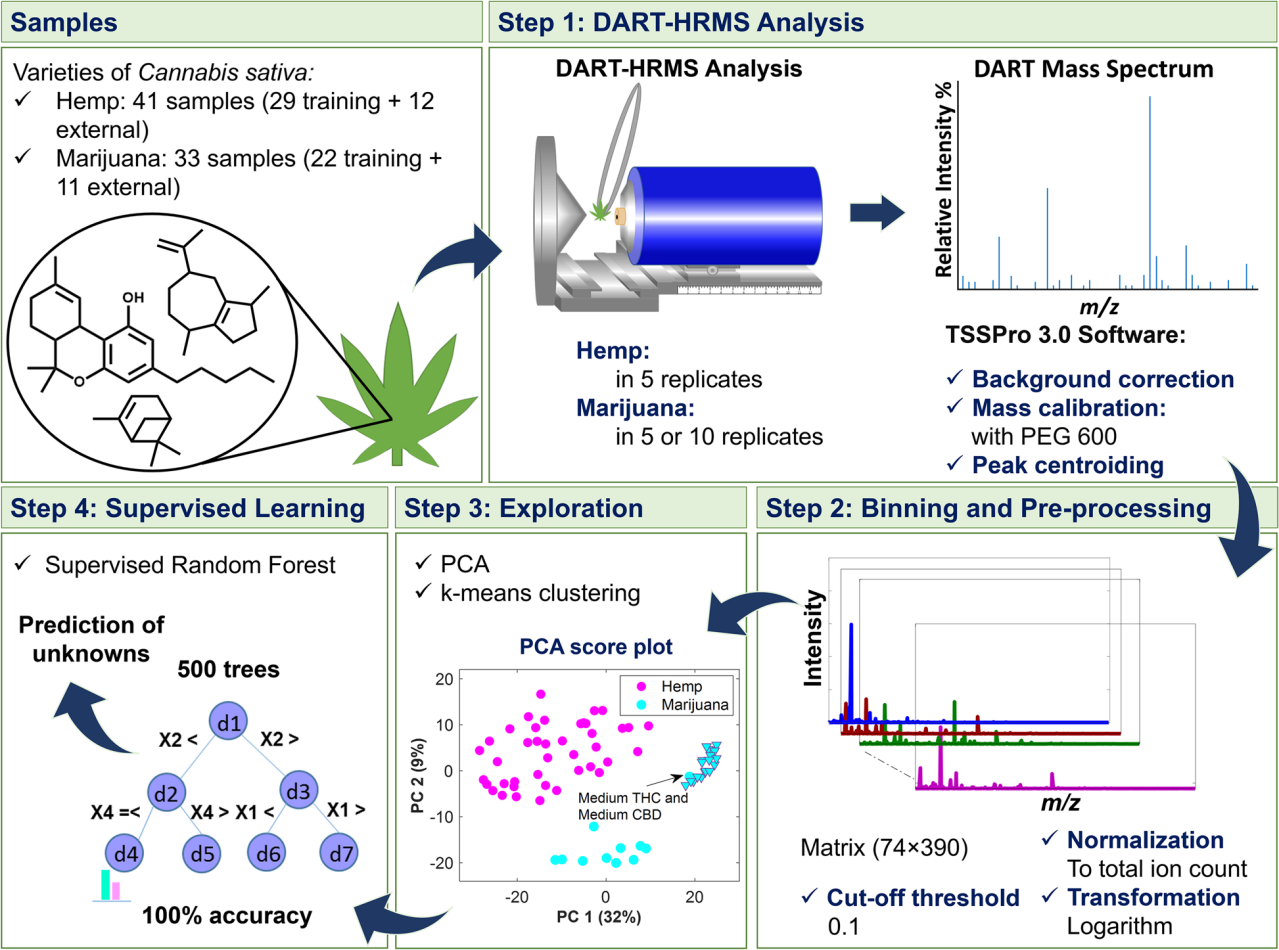
Workflow for discrimination of hemp and marijuana samples

The study presented here, which is summarized in the scheme presented in Fig. 1, utilized DART-HRMS, for the first time, to investigate the complex genome-defined chemical fingerprints of hemp and marijuana (with no sample pretreatment) for the purpose of distinguishing between these two *C. sativa* varieties using multivariate statistical approaches. Advanced chemometrics was applied to the DART-HRMS data derived from commercial hemp, recreational marijuana, and marijuana samples from Drug Enforcement Administration (DEA)-registered suppliers to develop a robust model by which they (i.e., hemp and marijuana) could be readily differentiated. The success rate of the developed model’s ability to predict external validation samples was 100%, indicating a high level of certainty. Importantly, the developed method circumvents the need to separate and differentiate cannabinoids by chromatography techniques (i.e., the traditional forensic approach for determining the THC concentration in a sample and which is used for differentiating between hemp and marijuana), in addition to bypassing all sample pretreatment steps.

## Materials and methods

### *Cannabis sativa* plant materials

Twenty-nine *C. sativa* flower samples of the hemp variety were purchased from three online vendors: (1) CBD Hemp Direct (Las Vegas, Nevada, USA), (2) Berkshire CBD (Brattleboro, Vermont, USA), and (3) Plain Jane (Berkeley, California, USA). These samples were used to build the model (i.e., training set). An additional 12 samples of hemp plant material were purchased from Plain Jane (Medford, Oregon, USA) at a later date to test the model (i.e., they were used for external validation). Additional information (e.g., cultivar/strain, vendor, batch number) for these hemp materials is provided (see Additional file 1).

*C. sativa* plant material of the marijuana variety was obtained from two DEA-registered sources. The National Institute on Drug Abuse (NIDA) (Research Triangle Park (RTP), North Carolina, USA) Drug Supply Program, which is part of the National Institutes of Health (NIH), provided the following four samples (i.e., cultivars) with varying levels of THC and cannabidiol (CBD) (the major non-psychoactive constituent in *C. sativa*): 1 g low THC cultivar (low THC/very high CBD), 1 g medium THC cultivar (medium THC/medium CBD), 1 g high THC cultivar (high THC/low CBD), and 1 g very high THC cultivar (very high THC/low CBD). The National Institute of Standards and Technology (NIST) (Gaithersburg, Maryland, USA) provided eight 0.5 g samples of marijuana that were confiscated by local law enforcement at different times over the past few years. Twenty-one strains of recreational marijuana were purchased from Garden Remedies Marijuana Dispensary (Melrose, Massachusetts, USA). Ten of the recreational samples were randomly selected for use in the development of the training model, while the remaining 11 samples were used to test the model (i.e., for external validation). Information for all marijuana samples (e.g., sample name, brand, supplier/vendor, batch number, etc.) is available (see Additional file 1).

### Mass spectral acquisition and analysis of DART-HRMS-derived data

The collection of mass spectral data was achieved by employing DART-HRMS. Two DART-HRMS instruments were used: (1) mass spectral data collected for all hemp products and the marijuana samples from DEA-registered suppliers were analyzed using the DART-HRMS instrument at the University at Albany (UAlbany) (Albany, New York, USA) and were translated and calibrated prior to data processing; and (2) all recreational marijuana flower samples were analyzed at IonSense Inc. (Saugus, Massachusetts, USA), with the raw data files calibrated, processed, and evaluated at UAlbany. The DART SVP (simplified voltage and pressure) ion source at IonSense was coupled to a JEOL AccuTOF high-resolution time-of-flight (TOF) mass spectrometer (Peabody, Massachusetts, USA) with a resolving power of 6000 full width at half maximum (FWHM) and mass accuracy of 5 millimass units (mmu). Data were collected in positive-ion mode using a DART ion source grid voltage of 300 V with the following mass spectrometer settings: ring lens, 5 V; orifice 1, 20 V; orifice 2 voltage, 5 V; peak voltage, 600 V; and detector voltage, 2000 V. The DART SVP ion source at UAlbany was also coupled to a JEOL AccuTOF high-resolution TOF mass spectrometer. The only difference between the DART ion source settings used at the two facilities was that the grid voltage at UAlbany was 250 V instead of 300 V. All mass spectral data were collected at a DART gas temperature of 350 °C using ultra-high purity helium gas at a flow rate of 2 L/min. Mass spectra were collected at a rate of 1 spectrum per second over a mass range of *m/z* 60–1000. TSSPro 3.0 software from Shrader Software Solutions (Grosse Pointe, Michigan, USA) was used for the calibration, spectral averaging, background subtraction, and peak centroiding of mass spectral data. Polyethylene glycol (PEG 600) (Sigma Aldrich, St. Louis, Missouri, USA) was used as the mass calibrant for all samples. Processing of the mass spectra of hemp and marijuana samples was performed with the Mass Mountaineer software suite from RBC Software (Portsmouth, New Hampshire, USA).

### Multivariate data analysis

The workflow which extended from DART-HRMS data collection to multivariate data analysis is displayed in Fig. 1. In Step 1, DART mass spectra of the *C. sativa* samples representing hemp and marijuana varieties were acquired. The spectra in the form of text files were imported into MATLAB 9.9.0, R2020b Software (The MathWorks, Inc., Natick, Massachusetts, USA) and R 3.5.1 (R Core Team 2018) for analysis. Each text file was comprised of a two-column matrix of *m/z* values and their corresponding abundances (i.e., ion counts). In Step 2, peaks were aligned along common *m/z* values by histogram estimation and nearest-neighbor correction methods using the “*mspalign*” function in MATLAB. The generated matrix contained the aligned spectra for the replicates of hemp and marijuana samples. The replicates for each sample were averaged, normalized, transformed (with log 10), and subjected to unsupervised (Step 3) and supervised analyses (Step 4). As shown in Step 3, PCA (Jolliffe and Cadima 2016) and k-means (Samut and Webb 2010; Lloyd 1982) were used to recognize the similarity and dissimilarity patterns of the samples and to reveal possible clusters, respectively. Silhouette width indexes were calculated to indicate the optimal number of clusters characterized by k-means and to validate the goodness of the clustering results. The data matrix was analyzed using supervised random forest (RF) (Liaw and Wiener 2001; Breiman 2001) (Step 4) to create a model for differentiating hemp and marijuana plant materials. RF is an ensemble of individual tree predictors, in which each tree in the forest is grown based on the independent replicas of training samples and variables. The samples not included in the replicates for a given tree (1/3 of the original dataset) are termed “out-of-bag” (OOB) for that tree. The overall accuracy and performance characteristics of the discrimination model were estimated based on the predictions of OOB observations and external validation samples.

## Results

### DART-HRMS analysis of *Cannabis sativa* plant material

**Fig. 2:**
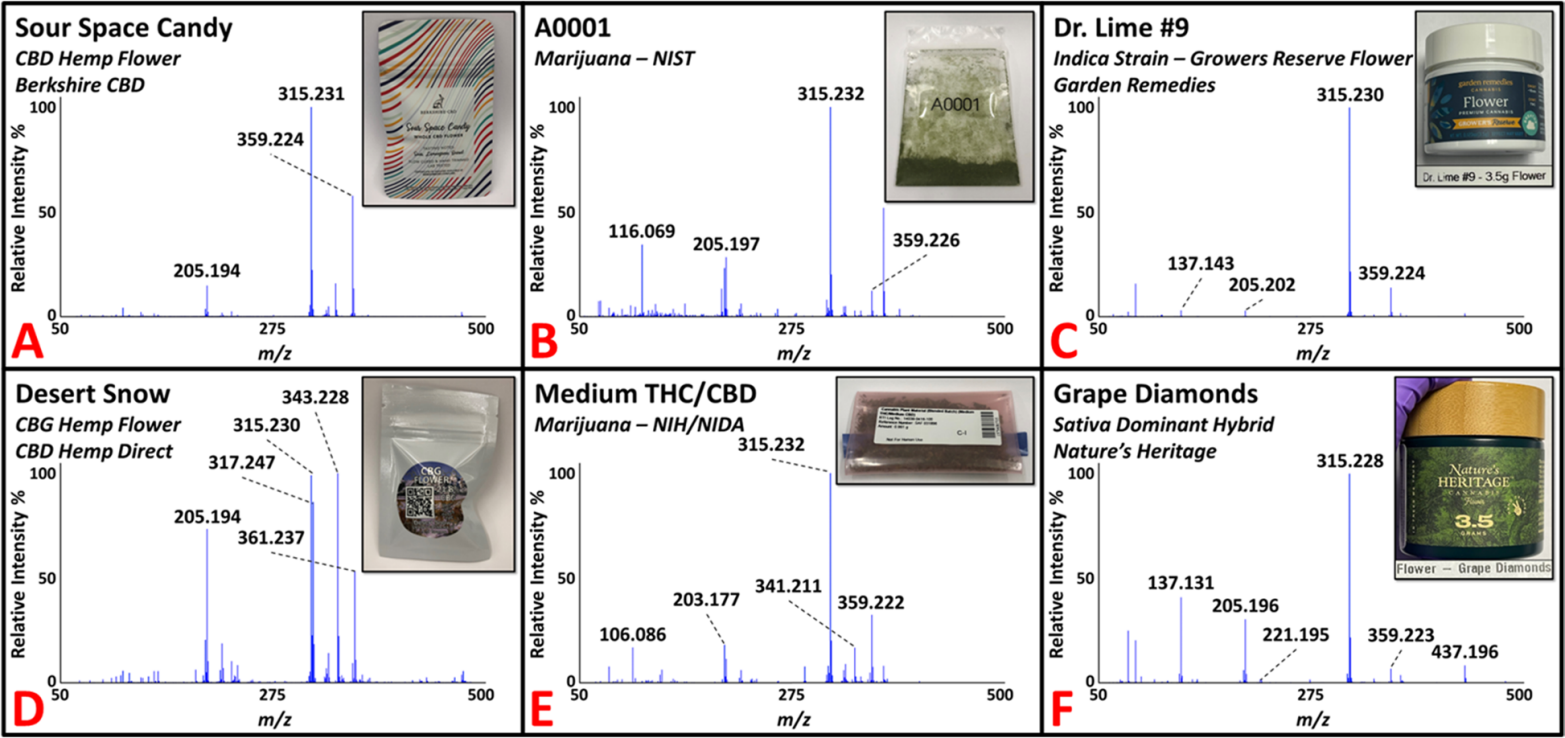
Representative DART-HR mass spectra of commercial hemp flower (panels **A** and **D**), marijuana samples supplied by NIST (panel **B**) and NIDA (panel **E**), and recreational marijuana flower products (panels **C** and **F**). Peaks consistent with the protonated masses of THC/CBD, CBG, THCA/CBDA, and CBGA at nominal *m/z* 315, 317, 359, and 361, respectively, were detected in the various samples

Initial investigations of *C. sativa* plant material focused on obtaining the DART-HRMS chemical profiles for both hemp and marijuana flower samples. Detailed information about the samples, including variety, cultivar/strain, vendor, and the batch number (when available) is provided (see Additional file 1). All samples were analyzed by inserting the closed end of a glass melting point capillary tube into the material and presenting the coated surface into the DART gas stream for approximately 5 s. A total of 29 hemp strains (i.e., cultivars) were purchased from three vendors at the beginning of this study, which included 27 CBD flower products and two cannabigerol (CBG) flower products. CBD flower contains high levels of CBD and cannabidiolic acid (CBDA), while CBG flower contains high levels of CBG and cannabigerolic acid (CBGA). An additional 12 hemp samples were purchased at a later date to test the developed model. Utilizing DART-HRMS is optimal for analyzing hemp and marijuana samples in their native forms (i.e., with no sample pretreatment, such as a decarboxylation step) to rapidly obtain the small-molecule profiles (i.e., in under one minute). The DART-HR mass spectra of all hemp flower samples (training-set hemp and test-set hemp) collected in positive-ion mode under soft ionization conditions (20 V) are available (see Additional file 2). Figure 2 shows representative DART-HR mass spectra acquired in positive-ion mode from analysis of *C. sativa* plant materials, including CBD (panel A) and CBG (panel D) hemp flower samples. The DART-HR mass spectra of all CBD hemp flower samples are very similar to one another; protonated masses consistent with CBD and CBDA were detected at *m/z* 315 and 359, respectively, in all samples. DART-HRMS analysis of the two CBG hemp flower samples also yielded these peaks, in addition to peaks at nominal *m/z* 317 and 361, which are consistent with the protonated masses of CBG and CBGA, respectively. The DART-HR mass spectra of the CBG hemp flower samples retained similarities with the CBD hemp flower profiles. However, indicative of the high CBG levels reported in the CBG flower samples, the relative intensities of the peaks attributed to CBG and CBGA were much higher in the DART-HR mass spectra of the CBG flower products.

*C. sativa* plant material of the marijuana variety was acquired from two U.S. DEA-registered sources: (1) NIDA supplied four marijuana samples (approximately 1 g each) through the NIDA/NIH Drug Supply Program; and (2) NIST provided eight marijuana samples (0.5 g each). All 12 marijuana samples were received in powdered form and were analyzed by DART-HRMS in positive-ion mode using the capillary tube sampling technique. Figure 2 presents two spectra of representative NIST (panel B) and NIDA (panel E) marijuana materials. Commercially available recreational marijuana samples were also analyzed. The DART-HR mass spectra for all marijuana samples from these suppliers are available (see Additional file 2). In total, 21 recreational marijuana samples were purchased from the Garden Remedies Marijuana Dispensary Adult-Use Menu. These products spanned the various marijuana strain types available (i.e., Indica-dominant, Sativa-dominant, hybrid), which represent *C. sativa* subspecies. Figure 2 presents two representative DART-HR mass spectra for Indica (panel C) and Sativa (panel F) dominant flower samples. The mass spectral profiles of all recreational marijuana flower products are available (see Additional file 2). Ten of the samples were randomly selected for inclusion in the training model. The remaining 11 recreational flower samples were used to test the prediction ability of the model (i.e., for external validation).

### Differentiation of hemp and marijuana varieties of *C. sativa*

**Fig. 3:**
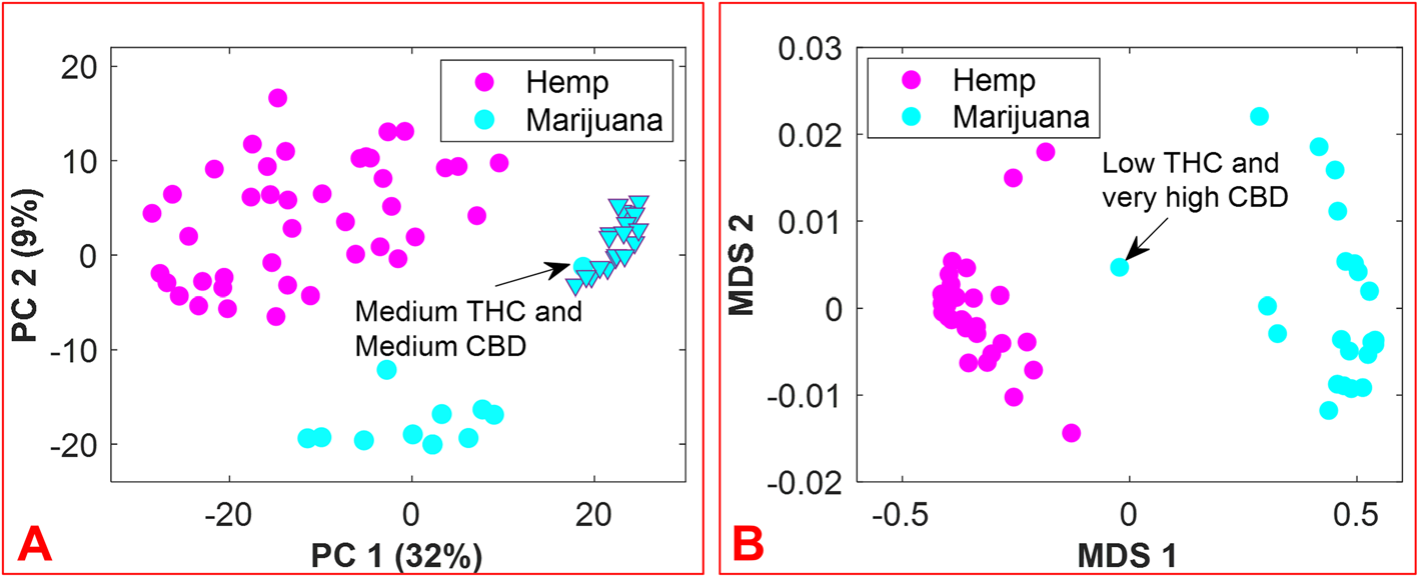
2D score plot resulting from PCA of hemp and marijuana sample spectra (panel **A**); 2D score plot of multidimensional scaling (MDS) analysis of the proximity matrix resulting from the application of supervised random forest (panel **B**). The magenta and cyan colors represent hemp and marijuana, respectively. The cyan triangles show the subset of recreational marijuana samples

The aim of this work was to accomplish the following: (1) develop a rapid, easy-to-use, and efficient means by which to differentiate hemp and marijuana varieties of *C. sativa*, and by extension, a method to identify *C. sativa* unknowns; and (2) circumvent some of the challenges typically encountered during the analysis of *C. sativa* materials when using chromatography-based methods. The approach is founded on the hypothesis that inherent in the small-molecule profiles of hemp and marijuana is the necessary information for the differentiation of these *Cannabis* varieties. Prior to the application of multivariate analysis methods to the features of the DART-HRMS-derived chemical profiles of hemp and marijuana, the spectra of all samples were binned to create a common *m/z* reference vector to ease their comparison. Accordingly, the “*mspalign*” function in MATLAB was performed with a hist resolution parameter of 0.01, while the peak relative abundance cutoff threshold was set to 0.1% of the maximum intensity to detect all potentially significant peaks. The marijuana samples provided by NIDA and NIST were packaged in plastic bags, the composition of which contributed to the DART-HRMS profiles of the samples. Thus, the *m/z* values derived from the packaging (e.g., nominal *m/z* 59, 75, 89, 107, 127) were removed from the data. Another *m/z* value that was removed was nominal *m/z* 371, which has been previously shown to be a plasticizer present on the capillary tubes used for sampling (Beyramysoltan et al. 2020). The resulting matrix had dimensions of 430 × 390 and contained the aligned spectra for the five replicates of each of the 41 hemp samples, the five replicates of each of the 21 recreational marijuana samples, and the 10 replicates of each of the 12 marijuana samples supplied by NIDA and NIST. The results of the preliminary PCA analysis were examined by Q residuals and Hotelling’s *T*^2^ statistic to detect any outliers, and this resulted in three spectra being removed from the data. Outlier spectra included those whose acquisition was accompanied by poor mass calibration or those that were not representative of a typical chemical profile. The averaging of sample replicates resulted in a matrix with dimensions of 74 × 390. Following logarithm transformation, the matrix was subjected to further analysis. Figure 3 panel A presents the PCA results as a 2-dimensional (2D) score plot, where the color-coded classes appear in the coordinate space represented by the first two principal components (PCs), which cover 41% of the data variance. While the recreational marijuana samples (cyan triangles) are located in close proximity to the NIDA-supplied marijuana sample that was reported to contain medium levels of both THC and CBD, they were distant from the other NIDA and NIST samples. These results support previous studies that indicated differences between marijuana sold at dispensaries, and that provided for research purposes by DEA-registered suppliers (Schwabe et al. 2021; Vergara et al. 2017). Clustering by k-means using one minus correlation metrics resulted in the categorization of the hemp samples into one cluster (magenta circles) and the marijuana samples into the other cluster (cyan circles).

Even though the DART-HR mass spectra of hemp and marijuana plant materials are readily visually apparent, a more objective approach to the assessment of the identity of *C. sativa* material was devised, using the random forest algorithm. This was applied to the 74 × 390 matrix. A total of 33 flower samples (12 hemps and 11 marijuana) of the 74 total *C. sativa* samples were randomly selected for external validation to examine the ability of the model to accurately predict the class assignments for new sample unknowns. The number of variables (which were randomly sampled as candidates at each split), and the number of trees found to be optimal were 20 and 500, respectively. Figure 3 panel B displays the proximity matrix generated from using supervised RF with a multidimensional scaling (MDS) method to show the pairwise similarities in a 2D Cartesian space, with the magenta and cyan points corresponding to the hemp and marijuana samples, respectively. It demonstrates the number of times that observations ended up in the same leaf node. According to Fig. 3 panel B, although the NIDA marijuana sample reported as low THC/very high CBD is located between the two groups, the samples belonging to each group are close together and separated from the samples of the other group.

**Fig. 4:**
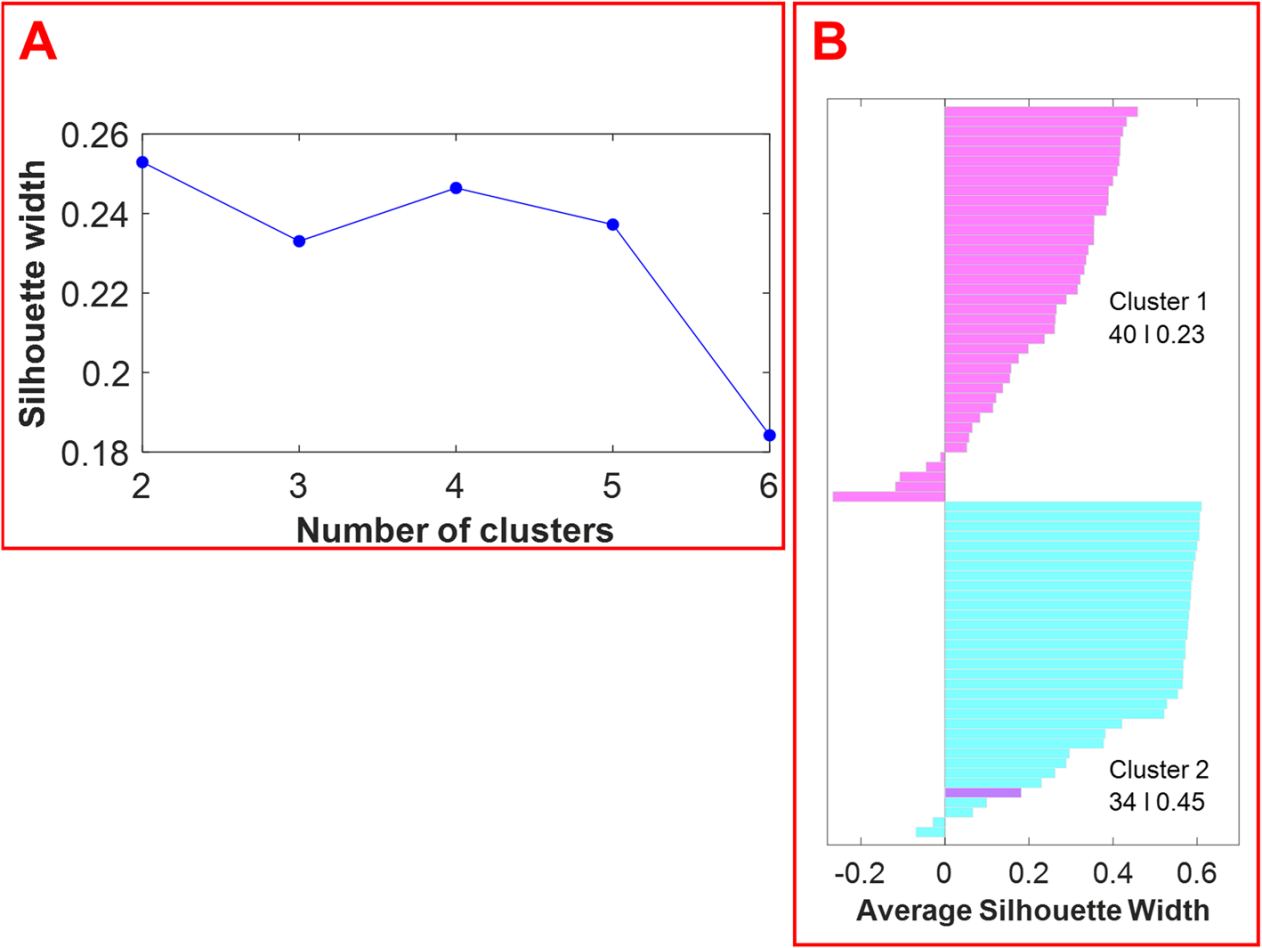
The average silhouette width over a range of cluster numbers (2–6) reveals that the optimum number of clusters is 2 (panel **A**). A silhouette plot (i.e., the visualization of the silhouette width for each sample) reveals the results with two clusters (panel **B**). Cluster 1 contains 40 members and cluster 2 contains 34 members. Hemp samples are shown in magenta, while marijuana samples are shown in cyan

The optimal number of clusters was estimated by computing the average silhouette (which measures the quality of the clustering) of observations for different numbers of clusters. Figure 4 panel A displays the average silhouette width over a range of the possible number of clusters. The optimal number of clusters is the one that maximizes the average silhouette width. Based on the information provided in Fig. 4 panel A, the optimal number of clusters is two. The silhouette plot in Fig. 4 panel B displays silhouette coefficients for each sample when the data are split into two clusters. The silhouette width of each sample is a measure of how similar each sample is to its respective cluster in comparison to the other cluster. As shown in Fig. 4, the optimum number of clusters is two: cluster 1 (magenta) has 40 members with a mean width of 0.23, and cluster 2 (cyan) has 34 members with a mean width of 0.45. Cluster 1 and cluster 2 members correspond to the samples of hemp and marijuana, respectively. One hemp sample was falsely clustered with the marijuana samples. The average silhouette width for the cluster of marijuana samples is higher than the average silhouette width for the hemp samples. This demonstrates that the cluster of marijuana samples is denser and that the samples are more similar to one another.

To reveal the model’s ability to distinguish between hemp and marijuana samples, Table 1 presents the confusion matrix for the prediction of OOB samples, while Table 2 contains the performance characteristics of the model (accuracy, sensitivity, specificity, and precision) for predicting the OOB samples. According to this table, the model performed well and the accuracy for predicting OOB samples is 98%.

**Table 1:** Confusion matrix associated with the prediction of “out-of-bag” samples in the random forest model

**Confusion matrix**		**Prediction**	
		**Hemp**	**Marijuana**
**True**	**Hemp (29)**	**1.00**	0.00
	**Marijuana (22)**	0.04	**0.96**

**Table 2:** Performance results of the random forest model for prediction of “out-of-bag” and external validation samples

	**Out-of-bag samples**		
	**Accuracy: 0.98 (98%)**		
	**Sensitivity**	**Specificity**	**Precision**
**Hemp (29)**	**1.00**	**0.96**	**0.97**
**Marijuana (22)**	**0.96**	**1.00**	**1.00**
	**External *C. sativa* plant materials**		
	**Accuracy: 1.00 (100%)**		
	**Sensitivity**	**Specificity**	**Precision**
**Hemp (12)**	**1.00**	**1.00**	**1.00**
**Marijuana (11)**	**1.00**	**1.00**	**1.00**

### Classification of external *C. sativa* plant materials

The remaining 11 recreational marijuana flower products that were not included in the training set, in addition to the 12 hemp products purchased after the model had been developed, were screened against the model to test its ability to classify samples that were unknown to the model. Table 3 shows the confusion matrix results for the prediction of the test samples (i.e., for external validation). In addition, Table 2 shows the performance characteristics of the model for predicting the external *C. sativa* samples, with all performance merits equal to 1 for both test sample sets (i.e., hemp and marijuana). The information presented in Tables 1, 2, and 3 reveal that the model is well-fitted for discriminating the two *C. sativa* varieties.

**Table 3:** Confusion matrix associated with the prediction of external validation samples using a random forest model

**Confusion matrix**		**Prediction**	
		**Hemp**	**Marijuana**
**True**	**Hemp (12)**	**1.00**	0.00
	**Marijuana (11)**	0.00	**1.00**

## Discussion

The most common methods for differentiating hemp and marijuana plant materials are chromatography-based approaches (e.g., GC-FID, GC–MS, HPLC–UV) (Pourseyed Lazarjani et al. 2020; UNODC 2009), with the categorization based upon THC content. Several reports have emphasized the use of GC-FID (Fischedick et al. 2010a; Zekič et al. 2020; Dussy et al. 2005; Fischedick et al. 2010b; Hazekamp et al. 2004; Hazekamp et al. 2012) and GC–MS (Zekič et al. 2020; Hazekamp et al. 2004, 2005; Namdar et al. 2018, 2019; Omar et al. 2013; Knight et al. 2010) methods for detection of natural cannabinoids (among other *Cannabis*-derived molecules) in various *Cannabis* plant materials. Modifications to standard GC-FID and GC–MS protocols include GC-vacuum UV (VUV) spectroscopy (Leghissa et al. 2018), two-dimensional GC-FID (GCxGC-FID) (Gröger et al. 2008), and GCxGC-MS with multivariate curve resolution-alternating least squares (MCR-ALS) (Omar et al. 2014). However, these methods rely upon the quantification of THC, which can be plagued with a number of analytical challenges, such as baseline separation of peaks and lengthy sample preparation protocols.

In an effort to circumvent the need to extend run times or incorporate extra sample preparation steps, several studies have investigated alternative sample collection techniques coupled with chromatography-based methods to differentiate *C. sativa* varieties. One study demonstrated the use of capillary microextraction of volatiles (CMV) coupled with GC–MS to distinguish the headspace volatiles of marijuana and hemp products based on their apparently distinct volatile organic compound (VOC) profiles (Wiebelhaus et al. 2016). However, this report revealed that potential adulterants and inconsistent packaging of samples may have contributed to the observed distinctions (Wiebelhaus et al. 2016). Another study utilized GC–MS coupled with dispersive pipette extraction (DPX) to investigate forensic casework marijuana and donated hemp samples (Horne et al. 2020). Although the approach was successful at differentiating the two varieties with greater than 98% accuracy, a significant reduction of THC stability after 48 h indicated that the samples would need to be reanalyzed if there was a delay between sample preparation and instrumental analysis (Horne et al. 2020). Another GC-based study sought to differentiate hemp and marijuana through their cannabinoid and terpene profiles using GC-FID and principal component analysis (PCA) (Pacula et al. 2016). This study, which included two recreational cultivars and three pharmacy *Cannabis* samples, successfully distinguished between the two *C. sativa* varieties (Pacula et al. 2016). In this case, expanding the sample source diversity could strengthen the ability of the model to classify a wider range of *Cannabis* samples. Another study applied PCA algorithms to quantitative data acquired from high-performance liquid chromatography-mass spectrometry (HPLC–MS) analysis of *Cannabis* plant materials (Fischedick et al. 2010a). This study identified several cannabinoids essential for differentiating between *Cannabis* strain types (Fischedick et al. 2010a) (i.e., strains within the marijuana variety) as opposed to specifically targeting the cannabinoids essential to differentiating *C. sativa* varieties (i.e., hemp and marijuana), which would be important for criminal justice purposes in the U.S. Although many of these investigations were successful at differentiating between hemp and marijuana varieties or strains, the methods are reliant upon chromatography and are therefore susceptible to the aforementioned delineated challenges that can arise using this technique (i.e., lengthy run times, column contamination, etc.).

Non-chromatographic approaches that circumvent the requirement to separate and/or differentiate between cannabinoids have also been investigated for distinguishing hemp and marijuana. A hand-held Raman spectrometer coupled with orthogonal partial least squares-discriminant analysis (OPLS-DA) tools proved successful in differentiating between the two *C. sativa* varieties (Sanchez et al. 2020). However, “real” forensic casework samples are rarely received in pristine form, and as such, the Raman approach is susceptible to interferences from various components that may be associated with the complex matrix and interfere with the Raman signal. Another study utilized advanced statistical modeling of nuclear magnetic resonance (NMR) spectroscopy and mass spectral data of *C. sativa* extracts, (Chen and de Boves Harrington 2019), which is unique in that it is typically difficult to utilize NMR for the analysis of complex matrices and mixtures. Although effective, this instrumentation is not commonly found in forensic or other *Cannabis* analysis laboratories due to expensive start-up and maintenance costs.

Colorimetric tests are also commonly used for differentiating between hemp and marijuana varieties of *Cannabis*, especially in forensic fieldwork, and these do not generally require instrumental analysis to arrive at a presumptive identification. A validated method utilizing the 4-aminophenol color test to differentiate hemp and marijuana revealed some degree of success (Lewis et al. 2021). However, this test can yield inconclusive results with samples that have THC and CBD levels that are within a factor of 3 of one another (Lewis et al. 2021). Another common color test for the identification of marijuana samples is the Fast Blue BB (FBBB) colorimetric test, which reacts with the cannabinoids present in *Cannabis* (primarily THC). A study utilizing this test found that hemp and marijuana plant materials could be classified correctly when linear discriminant analysis (LDA) was used to develop a model based on RGB (Red, Green, Blue) numerical codes from both fluorescence and color images that resulted from the application of the FBBB color test (Acosta and Almirall 2021). Positive-ion mode electrospray ionization Fourier transform-ion cyclotron resonance mass spectrometry (ESI( +)FT-ICR MS, ESI( +)MS/MS, ultraviolet–visible (UV–Vis) spectroscopy, and thin-layer chromatography (TLC) techniques have been used to investigate the products (i.e., chromophores) resulting from the application of the FBBB test to marijuana samples (dos Santos et al. 2016). In addition, direct analysis in real time-mass spectrometry (DART-MS) and ^1^H NMR techniques were coupled to identify the chromophores produced when various cannabinoids react with the FBBB reagent (França et al. 2020). A third color test to identify marijuana through the presence of THC is the Duquenois-Levine test. Research has been conducted to characterize (by mass spectrometry) the chromophores formed when cannabinoids react with the Duquenois reagents (Forrester 1997 Jacobs and Steiner 2014; Watanabe et al. 2016). Similar to the chromatography-based methods described, these tests all rely upon detection of THC specifically, which can complicate analyses because both marijuana and hemp contain this compound. Thus, while the distinction between marijuana and hemp has been defined based on THC levels, this is accompanied by the several aforementioned analytical challenges. By using the entire metabolomic profiles of hemp and marijuana acquired through ambient ionization mass spectrometry, the method presented here does not rely solely on the presence of any one molecule (or set of molecules), ratios of molecules to one another, or the ability to differentiate between cannabinoid isomers (i.e., THC and CBD).

The overall results of this study reveal that DART-HRMS yields consistent and unique chemical profiles for analyzed *Cannabis* materials that enable hemp and marijuana samples to be accurately differentiated, while circumventing challenges typically encountered with traditional chromatography methods (difficulties with cannabinoid separation and extensive sample preparation) and presumptive color tests (inconclusive or false positive results). Furthermore, this study utilized a sample set that demonstrates a balance between the total number of samples included, the number of replicates obtained, and a diversity in sources from which the *C. sativa* materials were acquired. This research provides a strong foundation upon which to develop a comprehensive mass spectral database for identifying unknown *C. sativa* variants through the acquisition of their DART-HR mass spectra. While the approach does not aim to replace confirmatory testing for THC concentrations, the model accomplishes the following: (1) bypasses the typical sample preparation steps required for analyzing materials by chromatography-based methods that seek to differentiate the samples through separation of their constituent cannabinoids; (2) reduces the chances for false positives that can result from presumptive color tests; and (3) serves as a supplementary tool for forensic investigators that enables more targeted confirmatory testing. This is timely and highly relevant, given the introduction in the U.S. House of Representatives of the “H.R.6645 – Hemp Advancement Act of 2022” bill (H.R.6645—117th Congress (2021–2022)). This act aims to amend the current federal ruling regarding hemp by: (1) changing the 0.3% [THC] designation to 1% and (2) replacing the word “delta-9” with the word “total” to include the various isomers of THC that have emerged in recent years (H.R.6645—117th Congress (2021–2022)). The introduction of this bill underscores some of the disadvantages of utilizing THC cutoffs in particular as the sole means by which to identify hemp and marijuana. Among other issues, it upends well-established and long-standing practices in criminalistics in a fashion that is expensive to address, since it will require the development of an entirely new set of protocols and data processing steps. Furthermore, it may not stand the test of time, as the cutoff thresholds are subject to change in the future. A method such as the one presented here, and which does not solely rely upon a 0.3% THC cutoff, is not at risk of becoming outdated upon further advancements of this bill or others in the U.S. House and Senate.

## Conclusions

A combined ambient ionization mass spectrometric (i.e., DART-HRMS) and chemometric approach was successfully used to create a prediction model that facilitated rapid high-accuracy differentiation of *C. sativa* hemp and marijuana plant materials obtained from multiple sources (i.e., commercial, DEA-registered, recreational). This method, which circumvents sample pretreatment steps (i.e., solvent extractions), addresses some of the difficulties encountered when analyzing samples using more conventional forensic analysis methodologies. A primary example of this is eliminating the need to separate and differentiate cannabinoids by chromatography techniques in order to determine the sample’s THC content, which is the primary basis for distinguishing between hemp and marijuana varieties of *Cannabis* for most methods. When new hemp and recreational marijuana flower products were screened against the model developed in this study, 100% accuracy in prediction was observed. The identities of *m/z* values that were determined to be important for the optimal differentiation of hemp and marijuana are the subject of continuing investigations. In addition, it is possible that *C. sativa* materials (of either the hemp or marijuana variety) with atypical levels of minor cannabinoids (such as CBN or isomers of THC) may respond differently in the DART gas stream and that this, in turn, may influence the results predicted by the model. Therefore, samples such as these will be investigated (as was done with the analysis of the two CBG hemp flower samples), along with new samples/strains from commercial and DEA-registered suppliers as they become available so that the model reflects ongoing changes in the chemical profiles of *Cannabis* products on the market.

## Supplementary Information

**Additional file 1.** Supplementary Mass Spectral Data and Sample Information. (1) Information about *C. sativa *plant materials analyzed in this study.

**Additional file 2.** Supplementary Mass Spectral Data for *C. sativa* Materials. (1) DART-HR mass spectra for hemp and marijuana materials.

## Data Availability

The datasets analyzed in the current study are available upon request at the discretion of the corresponding author.

## Abbreviations

2D: 2-Dimensional
CBD: Cannabidiol
CBDA: Cannabidiolic acid
CBG: Cannabigerol
CBGA: Cannabigerolic acid
CMV: Capillary microextraction of volatiles
DART: Direct analysis in real-time
DEA: U.S. Drug Enforcement Administration
DESI: Desorption electrospray ionization
DPX: Dispersive pipette extraction
ESI: Electrospray ionization
FBBB: Fast Blue BB
FID: Flame ionization detection
FT: Fourier transform
FWHM: Full width at half maximum
GBS: Genotyping-by-sequencing
GC: Gas chromatography
GCxGC: Two-dimensional gas chromatography
HR: High-resolution
HRMS: High-resolution mass spectrometry
HPLC: High-performance liquid chromatography
ICR: Ion cyclotron resonance
LDA: Linear discriminant analysis
MCD-ALS: Multivariate curve resolution-alternating least squares
MDS: Multidimensional scaling
mmu: Millimass unit
MS: Mass spectrometry
NIDA: National Institute on Drug Abuse
NIH: National Institutes of Health
NIJ: National Institute of Justice
NIST: National Institute of Standards and Technology
NMR: Nuclear magnetic resonance
OOB: Out-of-bag
OPLS-DA: Orthogonal partial least squares-discriminant analysis
PC: Principal component
PCA: Principal component analysis
PEG: Polyethylene glycol
PLS-DA: Partial least squares-discriminant analysis
RF: Random forest
RGB: Red, Green, Blue
RTP: Research Triangle Institute
SNP: Single-nucleotide polymorphism
SUNY: State University of New York
SVP: Simplified voltage and pressure
TD: Thermal desorption
THCA: Delta-9-tetrahydrocannabinolic acid
TLC: Thin-layer chromatography
TOF: Time-of-flight
UAlbany: The University at Albany
UV: Ultraviolet
Vis: Visible
VOC: Volatile organic compounds
VUV: Vacuum UV
∆^9^-THC or THC: Delta-9-tetrahydrocannabinol
