# Supplementary material for: Combined ambient ionization mass spectrometric and chemometric approach for the differentiation of hemp and marijuana varieties of Cannabis sativa
Source: J Cannabis Res. 2023 Feb 18;5:5. doi: 10.1186/s42238-023-00173-0 (PMC9938564; doi:10.1186/s42238-023-00173-0)
Supplement: Supplementary file 1 — Additional file 1. Supplementary Mass Spectral Data and Sample Information. (1) Information about C. sativa plant materials analyzed in this study. [file 42238_2023_173_MOESM1_ESM.docx]

**Combined Ambient Ionization Mass Spectrometric and Chemometric Approach for the Differentiation of Hemp and Marijuana Varieties of *Cannabis sativa***

AUTHORS: Megan I. Chambers,^a^ Samira Beyramysoltan,^a^ Benedetta Garosi^a^ and Rabi A. Musah^a^*

^a^Department of Chemistry, University at Albany – State University of New York (SUNY), 1400 Washington Avenue, Albany, NY 12222, United States

*Corresponding author: rmusah@albany.edu

**Supporting Information: *Cannabis sativa* Sample/Product Information**

| **Table S1**. Information on the analyzed hemp samples including *C. sativa* variety, cultivar/strain, vendor, vendor abbreviation, and batch number. | | | |
| --- | --- | --- | --- |
| ***C. sativa* Variety** | **Cultivar/Strain** | **Vendor*** | **Batch Number** |
| Hemp | Acapulco Gold | CBD Hemp Direct | 1912NVC2044-11643 |
| Hemp | AVW1 | Plain Jane (OR) | **-** |
| Hemp | Berry Blossom | Plain Jane (OR) | **-** |
| Hemp | Casino Cookies | CBD Hemp Direct | 001CC |
| Hemp | Casino Cookies #2 | CBD Hemp Direct | 1909NVC1598-8477 |
| Hemp | Cat’s Meow | Plain Jane (OR) | **-** |
| Hemp | Charlotte's Sauce #2 | CBD Hemp Direct | 1908NVC1326-6578 |
| Hemp | Desert Snow CBG | CBD Hemp Direct | 1910NVC1598-8476 |
| Hemp | Durban Potion #2 | CBD Hemp Direct | 002DP |
| Hemp | Early Blossom | Plain Jane (OR) | **-** |
| Hemp | Elektra | Berkshire CBD | NAB021900012 |
| Hemp | Elektra | Plain Jane (CA) | **-** |
| Hemp | GSC | CBD Hemp Direct | **-** |
| Hemp | Harle Tsu | Plain Jane (OR) | **-** |
| Hemp | Hemp World Haze | CBD Hemp Direct | 1909NVC1403-7044 |
| Hemp | Honolulu Haze #2 | CBD Hemp Direct | 1909NVC1403-7044 |
| Hemp | Jack Herer | Plain Jane (OR) | **-** |
| Hemp | Jazzy CBD #2 | CBD Hemp Direct | 002JZ |
| Hemp | Jazzy CBG | CBD Hemp Direct | 1910NVC1598-8479 |
| Hemp | Juicy Fruit | CBD Hemp Direct | 001JF |
| Hemp | Juicy Fruit #2 | CBD Hemp Direct | 1909NVC1598-8478 |
| Hemp | Lifter | Berkshire CBD | NAB021900010 |
| Hemp | Lifter | Plain Jane (CA) | **-** |
| Hemp | Paradise OG | CBD Hemp Direct | 001POG |
| Hemp | Pineapple Tsunami | Plain Jane (OR) | **-** |
| Hemp | Skywalker | Plain Jane (OR) | **-** |
| Hemp | Special Sauce | Berkshire CBD | NAB02190003 |
| Hemp | Special Sauce | Plain Jane (CA) | **-** |
| Hemp | Sour Space Candy | Berkshire CBD | NAB02190005 |
| Hemp | Stormi D | Plain Jane (OR) | **-** |
| Hemp | Sunset Rd. Sherbert | CBD Hemp Direct | 001SR |
| Hemp | Sunset Rd. Sherbert #2 | CBD Hemp Direct | 1909NVC1598-8480 |
| Hemp | Suver Haze | Berkshire CBD | NAB021900011 |
| Hemp | Suver Haze | Plain Jane (CA) | **-** |
| Hemp | Swiss Harlequin | Plain Jane (OR) | **-** |
| Hemp | T-1 | Plain Jane (OR) | **-** |
| Hemp | Tangie | CBD Hemp Direct | 001TG |
| Hemp | Trap Star | CBD Hemp Direct | 1908NVC1326-6579 |
| Hemp | Trophy Wife | CBD Hemp Direct | 001TW |
| Hemp | Vegas Lights #2 | CBD Hemp Direct | 1910NVC1598-8481 |
| Hemp | Wife Reserve | Plain Jane (OR) | **-** |
| *Plaine Jane products were obtained from California (CA) or Oregon (OR) | | | |

| **Table S2.** Information on the analyzed marijuana samples including *C. sativa* variety, sample name, supplier, and batch number. | | | |
| --- | --- | --- | --- |
| ***C. sativa* Variety** | **Sample Name** | **Supplier** | **Batch Number** |
| Marijuana | A0001 | NIST | **-** |
| Marijuana | A0007 | NIST | **-** |
| Marijuana | A0009 | NIST | **-** |
| Marijuana | A0027 | NIST | **-** |
| Marijuana | A0076 | NIST | **-** |
| Marijuana | A0096 | NIST | **-** |
| Marijuana | A0102 | NIST | **-** |
| Marijuana | A0115 | NIST | **-** |
| Marijuana | High THC/Low CBD | NIDA | Barrel # 1291 |
| Marijuana | Low THC/Very High CBD | NIDA | Barrel # 1371 |
| Marijuana | Medium THC/Medium CBD | NIDA | Blended Batch |
| Marijuana | Very High THC/Low CBD | NIDA | Barrel # 1449F |

| **Table S3.** Information on the recreational marijuana samples including *C. sativa* variety, cultivar/strain, vendor, brand, strain-type (if applicable), additional information, and batch number (when available). | | | | | | |
| --- | --- | --- | --- | --- | --- | --- |
| ***C. sativa* Variety** | **Cultivar/ Strain** | **Vendor** | **Brand** | **Strain-Type** | **Additional Information** | **Batch Number** |
| Marijuana | Airplane Glue #3 | Garden Remedies | Garden Remedies | Hybrid | Special Stash Flower | B-1412-F2-1.5.22-04118 |
| Marijuana | APEX | Garden Remedies | Garden Remedies | **-** | Rollers Series Flower | B-1209-F9-06123-7.14.21 |
| Marijuana | Big D Energy | Garden Remedies | Garden Remedies | Sativa | Growers Reserve Flower | B-1355-F5-11.9.21-04014 |
| Marijuana | Cease and Desist | Garden Remedies | Garden Remedies | Hybrid | Growers Reserve Flower | B-1430-F9-1.17.22-08006 |
| Marijuana | Citrus Rain | Garden Remedies | Garden Remedies | Hybrid | Growers Reserve Flower | B-1337-F2-11.1.21-04607 |
| Marijuana | Crockett’s Haze | Garden Remedies | Green Harbor | **-** | **-** | CRHZFL222301/H03 |
| Marijuana | Deadfire OG | Garden Remedies | Garden Remedies | Hybrid | Growers Reserve Flower | **-** |
| Marijuana | Deadhead OG | Garden Remedies | Garden Remedies | Hybrid | Growers Reserve Flower | B-1407-F3-12.28.21-04399 |
| Marijuana | Dr. Lime #9 | Garden Remedies | Garden Remedies | Indica | Growers Reserve Flower | B-1408-F3-12.28.21-04396 |
| Marijuana | Dr. Lime #10 | Garden Remedies | Garden Remedies | Sativa | Growers Reserve Flower | B-1260-F11-06295-8.17.21 |
| Marijuana | Flavah-ICE | Garden Remedies | Garden Remedies | Sativa | Growers Reserve Flower | B-1452-F10-H-C-1.31.22-08209 |
| Marijuana | Grape Diamonds | Garden Remedies | Nature’s Heritage | Hybrid | Sativa-dominant | Grape Diamonds H2.8.22 F8 B4 T3 |
| Marijuana | Inzane in the Membrane | Garden Remedies | Garden Remedies | Sativa-dominant | Premium Flower | B-1258-F11-06286-8.16.21 |
| Marijuana | Lemon Peels | Garden Remedies | Garden Remedies | **-** | Growers Reserve Flower | B-1239-F10-06207-7.26.21 |
| Marijuana | Lilac Diesel | Garden Remedies | Solar Therapeutics | Hybrid | **-** | 2021-12-18-LD-H18-2 |
| Marijuana | Project Z | Garden Remedies | RiverRun Gardens | Sativa | **-** | **-** |
| Marijuana | Rainmaker | Garden Remedies | Garden Remedies | Hybrid | Growers Reserve Flower | B-1318-F8-10.13.21-06704 |
| Marijuana | Sherb Valley | Garden Remedies | The Heirloom Collective | **-** | **-** | F2H11SHER101921 |
| Marijuana | Snowdog | Garden Remedies | Berkshire Roots | Hybrid | **-** | **-** |
| Marijuana | Tropical Runtz | Garden Remedies | RiverRun Gardens | Sativa | **-** | **-** |
| Marijuana | Wonka Bars | Garden Remedies | Nature’s Heritage | Indica | **-** | Wonka Bars #7 H2.1.22 F11 B1 T3 |
