# Supplementary material for: Combined ambient ionization mass spectrometric and chemometric approach for the differentiation of hemp and marijuana varieties of Cannabis sativa
Source: J Cannabis Res. 2023 Feb 18;5:5. doi: 10.1186/s42238-023-00173-0 (PMC9938564; doi:10.1186/s42238-023-00173-0)
Supplement: Supplementary file 2 — Additional file 2. Supplementary Mass Spectral Data for C. sativa Materials. (1) DART-HR mass spectra for hemp and marijuana materials. [file 42238_2023_173_MOESM2_ESM.docx]

**Combined Ambient Ionization Mass Spectrometric and Chemometric Approach for the Differentiation of Hemp and Marijuana Varieties of *Cannabis sativa***

AUTHORS: Megan I. Chambers,^a^ Samira Beyramysoltan,^a^ Benedetta Garosi^a^ and Rabi A. Musah^a^*

^a^Department of Chemistry, University at Albany – State University of New York (SUNY), 1400 Washington Avenue, Albany, NY 12222, United States

*Corresponding author: rmusah@albany.edu

**Supporting Information: Mass Spectral Data for *C. sativa* Materials**


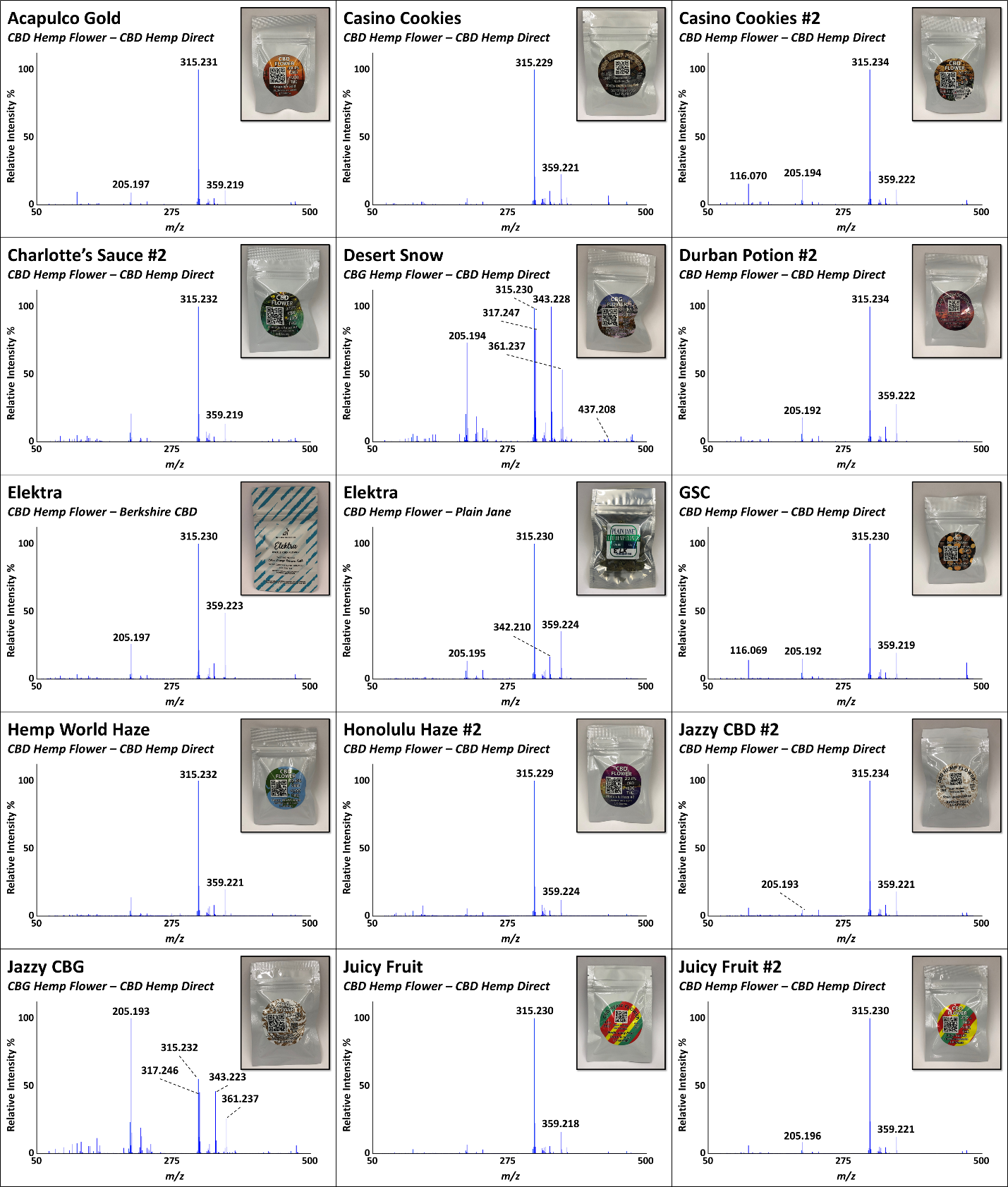


**Figure S1.** Representative DART high-resolution mass spectra of hemp samples used in the training set analyzed in positive-ion mode at 20 V. Images of the corresponding products in their packaging are shown in the insets.


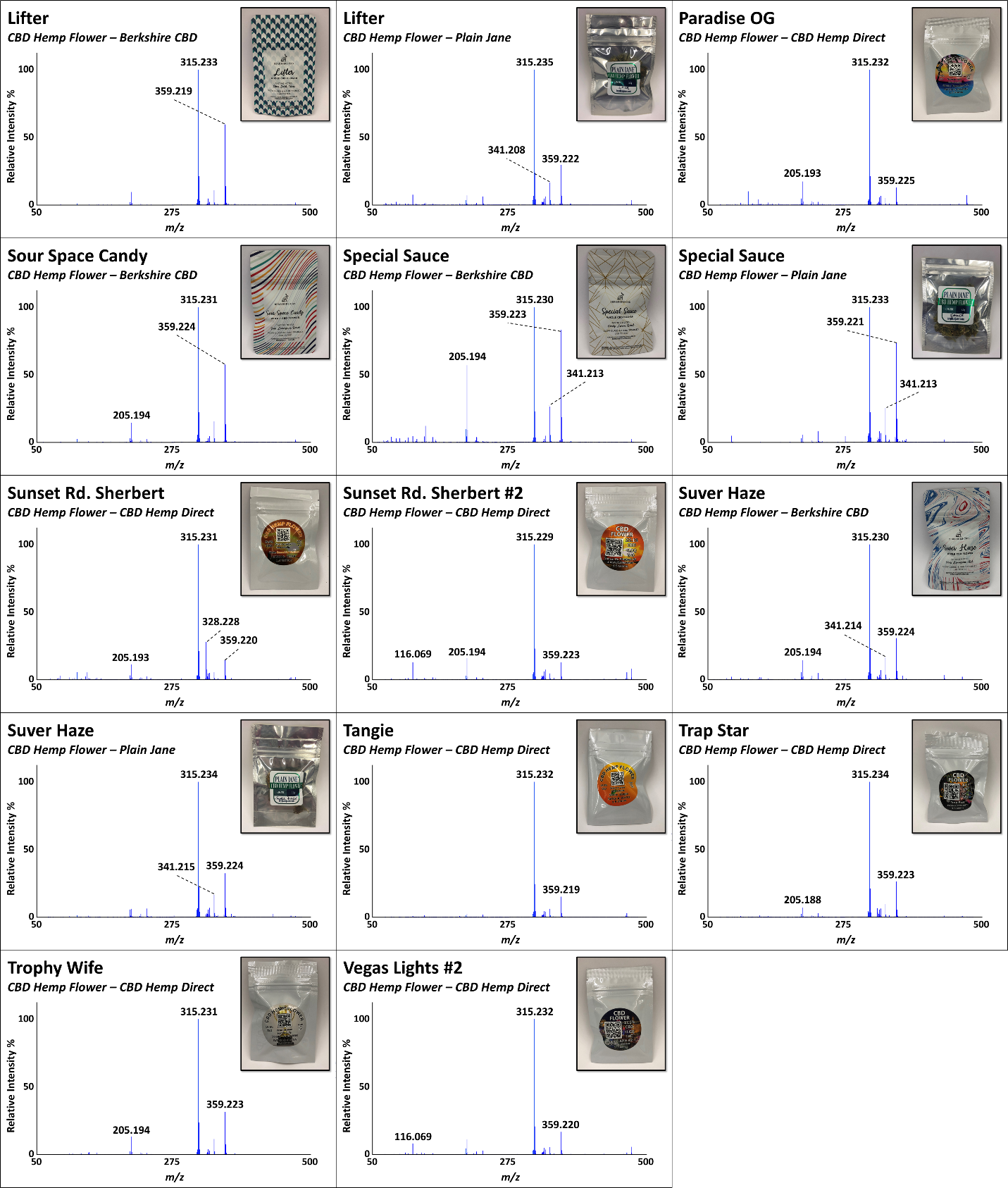


**Figure S1 (continued).** Representative DART high-resolution mass spectra of hemp samples used in the training set analyzed in positive-ion mode at 20 V. Images of the corresponding products in their packaging are shown in the insets.


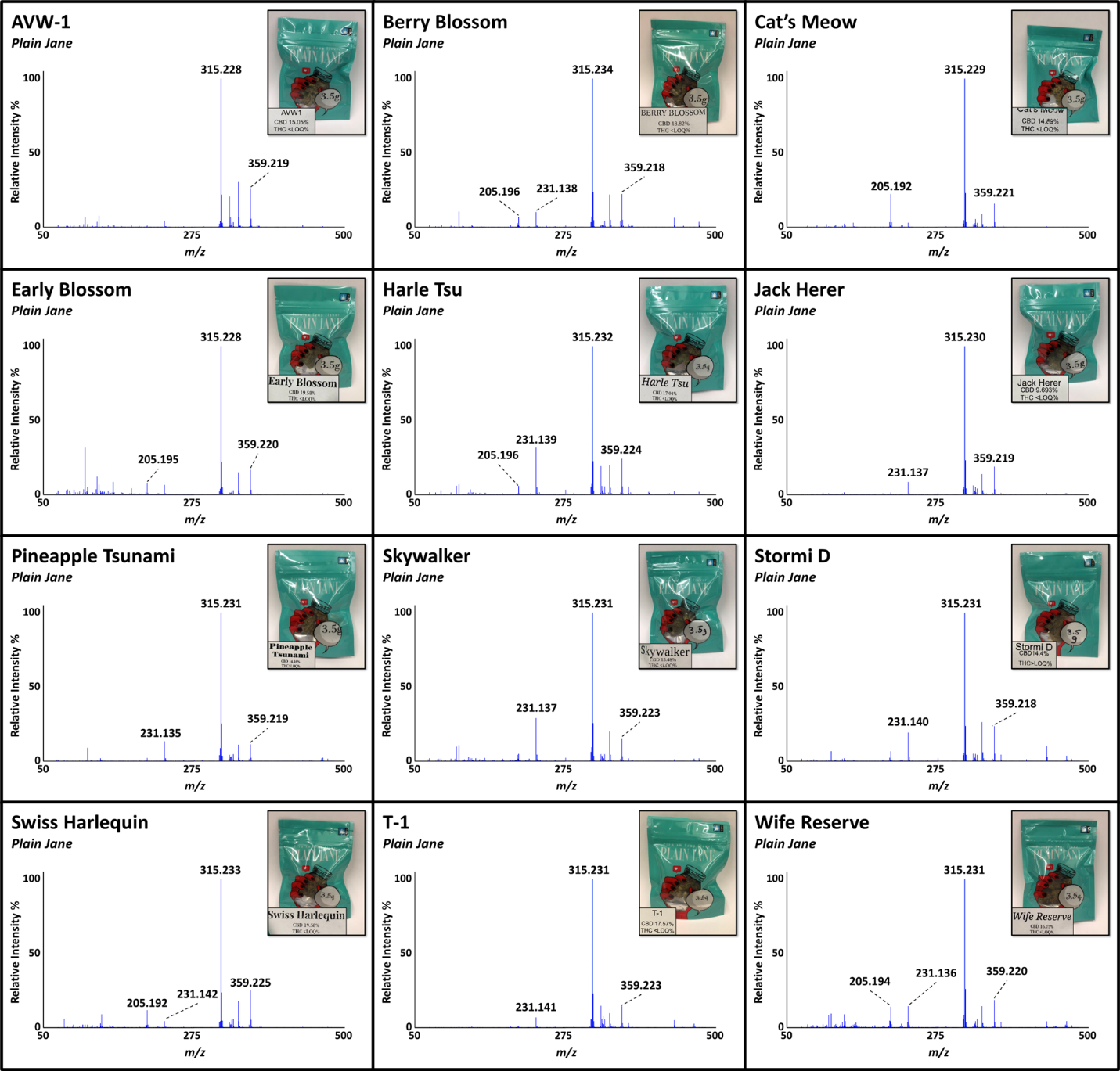


**Figure S2.** Representative DART high-resolution mass spectra of hemp flower samples from a commercial hemp vendor analyzed in positive-ion mode at 20 V. These samples were used to test the ability of the model to classify “unknowns”. Images of the corresponding packaging are shown in the insets.


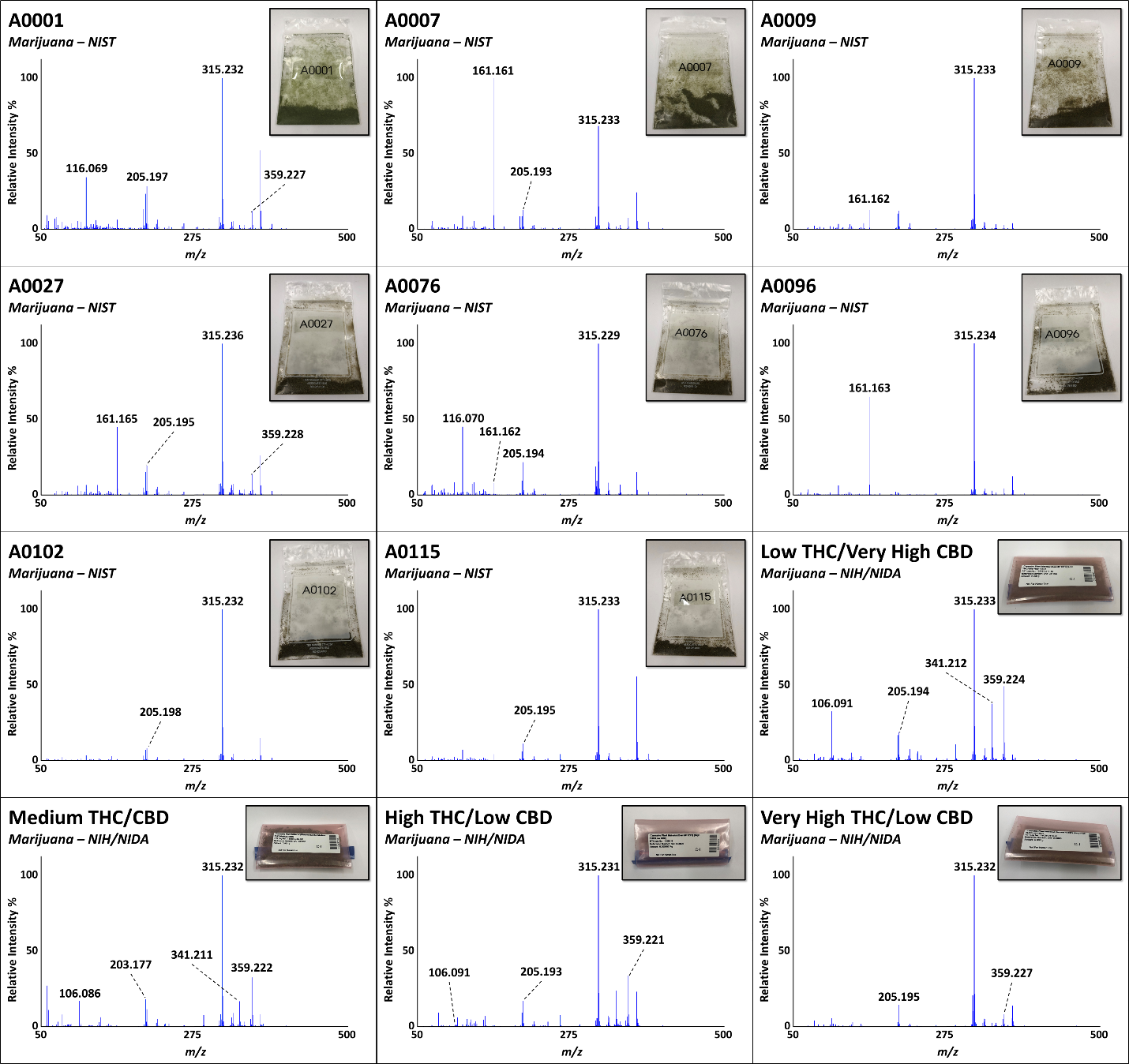


**Figure S3.** Representative DART high-resolution mass spectra of marijuana samples from DEA-registered suppliers analyzed in positive-ion mode at 20 V. Images of the corresponding packaging are shown in the insets.


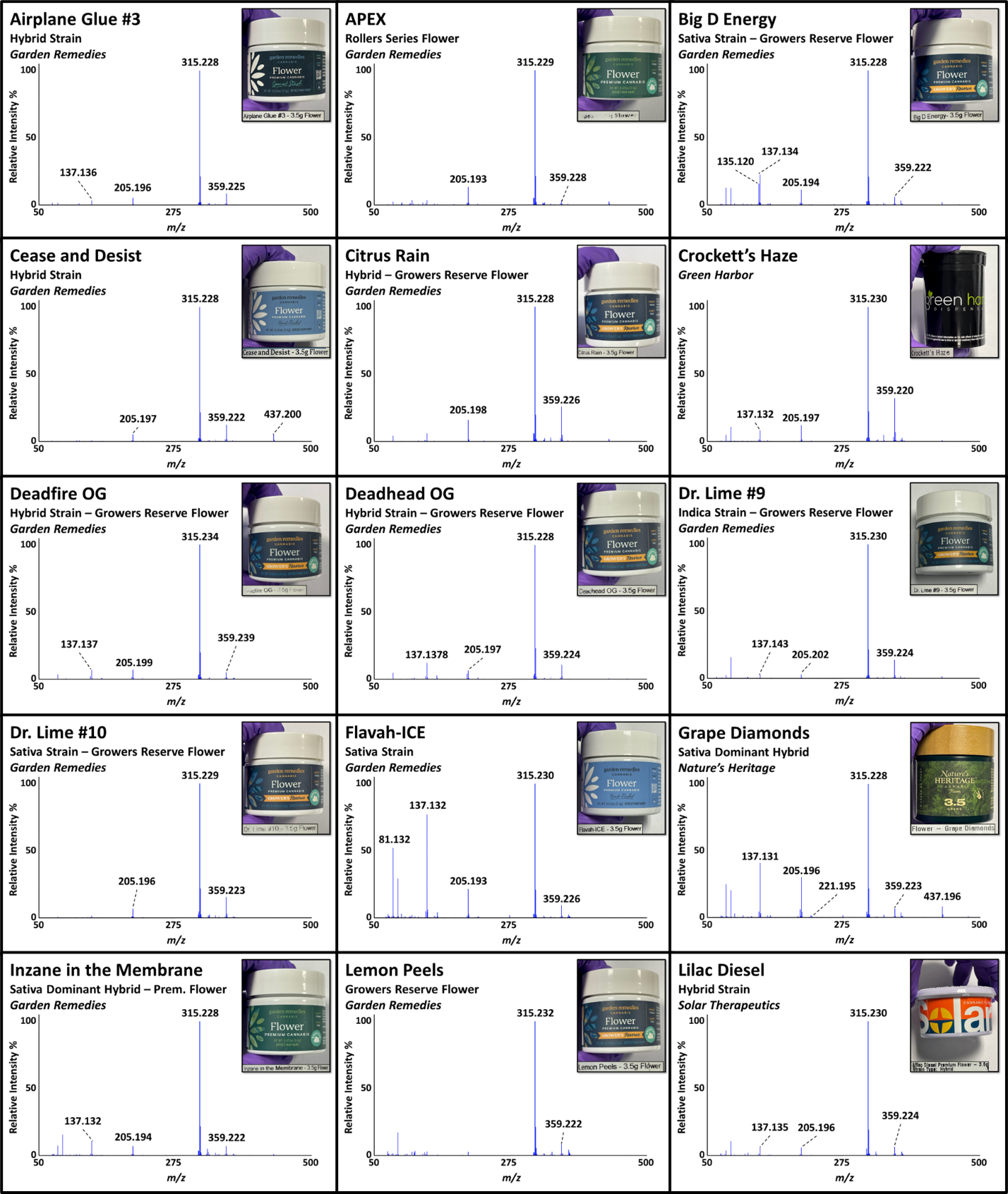


**Figure S4.** Representative DART high-resolution mass spectra of marijuana flower samples from a recreational *Cannabis* dispensary analyzed in positive-ion mode at 20 V. Images of the corresponding packaging are shown in the insets.


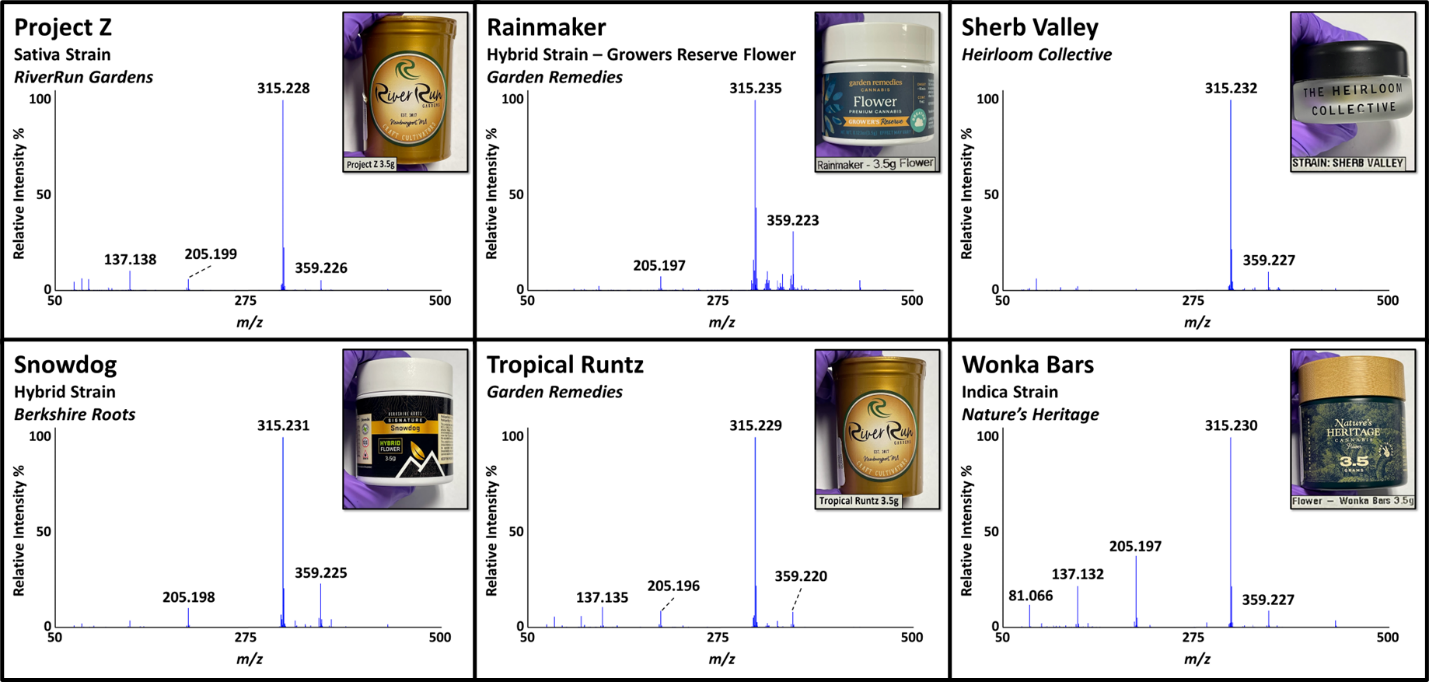


**Figure S4 (continued).** Representative DART high-resolution mass spectra of marijuana flower samples from a recreational *Cannabis* dispensary analyzed in positive-ion mode at 20 V. Images of the corresponding packaging are shown in the insets.
